# Health-Related Quality of Life in Gilles de la Tourette Syndrome: A Decade of Research

**DOI:** 10.3233/BEN-120296

**Published:** 2013

**Authors:** Andrea Eugenio Cavanna, Kate David, Valentina Bandera, Cristiano Termine, Umberto Balottin, Anette Schrag, Caroline Selai

**Affiliations:** ^1^Sobell Department of Movement DisordersInstitute of Neurology and University College LondonLondonUK; ^2^Department of NeuropsychiatryBSMHFT and University of BirminghamBirminghamUK; ^3^Child Neuropsychiatry UnitDepartment of Experimental MedicineUniversity of InsubriaVareseItaly; ^4^Department of Child Neurology and PsychiatryIRCCS 'C. Mondino' FoundationUniversity of PaviaPaviaItaly; ^5^Department of NeurologyRoyal Free and University College Medical SchoolLondonUK

**Keywords:** Gilles de la Tourette syndrome, health-related quality of life, functional impairment, tics, behavioural problems

## Abstract

Gilles de la Tourette syndrome (GTS) is a neurodevelopmental condition characterised by multiple motor and phonic tics and associated behavioural problems, carrying a significant burden on patients' lives. Although the term health related-quality of life (HR-QOL) has only been used in recent years, several studies have long addressed the impact of GTS on physical, psychological and social aspects of wellbeing of both children and adults with GTS. We set out to answer the question "Is HR-QOL affected by GTS and, if so, in what domains?" by conducting a systematic literature review of published original studies addressing HR-QOL in both children and adult patients with GTS. This review focuses on the current evidence on the impact of GTS on patients' lives, mainly informed by studies using generic functional impairment and HR-QOL measures from the last decade, and expands on the new opportunities introduced by the recently developed GTS-specific HR-QOL scales (GTS-QOL and GTS-QOL-C&A). Analysis of the first decade of studies specifically addressing HR-QOL in GTS suggests that co-morbid conditions are key factors in determining HR-QOL in young patients, whilst the picture is more complex in adults with GTS. These findings offer some general directions for both current clinical practice and future research.

## 1. The functional impact of Gilles de la Tourette syndrome across the lifespan

Gilles de la Tourette syndrome (GTS) is a life-long neurodevelopmental disorder defined by the presence of multiple motor and phonic tics [1]. Tics are repetitive, uncontrollable movements or vocalizations that usually present in childhood but may change with regard to their location, frequency and severity over time [2]. GTS affects approximately 1% of school-age youngsters and is three to four times more common in males than females [3]. GTS is increasingly recognised as a complex disorder, with a wide spectrum of associated behavioural problems that can accompany the motor and phonic tics. These problems include tic-related symptoms (ranging from socially inappropriate statements to actual self-harm) and co-morbid psychiatric disorders, such as obsessive-compulsive disorder (OCD) and attention-deficit hyperactivity disorder (ADHD) [4,5]. Large studies conducted both in clinical populations [2,6] and in the community [7] have consistently found that only about 10% of individuals with GTS do not present with behavioural co-morbidities.

ISSN 0953-4180/13/$27.50 © 2013 – IOS Press and the authors. All rights reserved

Given the clinical complexity of GTS, it is not surprising that its impact would extend beyond physical disability. In an early study examining functional impairment in patients with GTS, Stefl found that 53% of the participants had sought psychotherapy to assist them in coping with the impact of their tics on daily functioning and a number of related problems, including stigma, anxiety and depression [8]. This study first showed that co-morbid behavioural and emotional problems are often the primary incentive for seeking treatment. In a subsequent study, 88% of the sample reported that tics negatively influenced their daily functioning [9]. It has long been reported that patients with GTS experience distress in coping with tics in terms of disruption of daily activities [10,11], as well as difficulties with socializing [12]. The chronic presence of tics and tic-associated symptoms has been shown to affect both children and adults with GTS.

Young patients with GTS often report functional impairments, defined as the inability to perform routine and age-appropriate tasks in the domains of school, home, and social activities [13,14]. It has been suggested that a comprehensive assessment of GTS-related difficulties in young patients with GTS should take into account both self- and proxy-reports, which can differ substantially [15]. A study of 138 youths aged 5 to 18 years showed that 46% of children with GTS demonstrate school-related problems. Of note, patients with co-morbid ADHD had a nearly 4-fold increased risk for academic difficulty compared to those without ADHD [16]. Consistent findings have shown that much of the psychosocial and behavioural dysfunction in children with tic disorders appears to be a consequence of co-morbid ADHD, which is highly associated with disruptive behaviour and functional impairment [17–20]. Hoekstra et al. investigated social and behavioural problems related to ADHD, OCD and tic severity in children with a tic disorder [21]. In this study, the parents of 58 children diagnosed with a tic disorder with and without different forms of ADHD were asked to complete measures of behavioural problems. Patients with a tic disorder with ADHD-hyperactive/impulsive subtype had the highest questionnaire scores, subjects with ADHD-inattentive subtype had medium scores and subjects without ADHD had the lowest scores. The authors concluded that in patients with tic disorders, the presence and severity of ADHD are the main predictors of behavioural and social problems.

Some problems can become exacerbated across the lifespan. Adults with moderate-to-severe GTS attract public attention due to the sometimes bizarre nature of the combination of tics and behavioural symptoms. Since diagnosis is often made years after the onset of symptoms, patients with GTS cope with disruptive, uncontrolled symptoms without understanding their cause and often feel different from their peer group [22]. Moreover, most people with GTS try to camouflage or suppress their tics, at the expense of mounting inner tension, indicating a high degree of self-consciousness. Thibert et al. reported that adults who suffer from tics and co-morbid obsessive-compulsive symptoms have altered 'self-concepts' and self-esteem and increased social anxiety [23]. According to these authors' experience, accepting and adapting to obvious tic symptoms is more difficult for adults with co-morbid obsessive-compulsive symptoms, who tend to be perfectionist and more preoccupied with their own shortcomings.

Adults with GTS also report higher rates of unemployment and lower income, since employment status largely depends on potential employers' attitudes and other aspects of the social and cultural environment [24]. Furthermore, a high proportion of patients with GTS report problems in cognitive functioning (memory, concentration), which might explain poor academic achievement and therefore limited career choice, despite lack of evidence for major neuropsychological impairment [25]. Overall, it has been shown that patients with GTS tend to belong to a lower social class than their parents [26].

## 2. Studies of health related-quality of life in Gilles de la Tourette syndrome populations

Health related-quality of life (HR-QOL) has become increasingly relevant to both clinical practice (as a tool for the identification of care needs) and research (as a patient-reported outcome measure). HR-QOL is defined as the perceived physical, mental and social effects of an illness and associated therapies on a patient over time [27], as opposed to the generic concept of functional impairment.

GTS has been shown to have a significant impact on a patient's HR-QOL, particularly the widespread effect across social and emotional domains [28]. By definition, HR-QOL is a subjective parameter and consequently difficult to measure and quantify, however there is a need for such a measurement when developing and assessing the efficacy of interventions. Recent studies on the effectiveness of neurosurgical procedures for severe, treatment-resistant GTS have highlighted the importance of focusing on HR-QOL as a measure of overall patient satisfaction in outcome analysis. Relying solely on changes in tic severity does not always reflect the patient's overall well-being following neurosurgical interventions. For instance, when exploring the effectiveness of infrathalamic and thalamic lesioning for patients with intractable GTS, Babel, et al. found that a reduction in tic severity did not correlate with improved patient satisfaction [29]. More recently, Foltynie et al. showed that deep brain stimulation (DBS) of the motor portion of the globus pallidus – pars interna (GPi) may result in resolution of tics without a corresponding improvement in HR-QOL, highlighting the importance of behavioural and psychological factors [30]. Both studies advocated the use of a disease-specific measure of HR-QOL as a surgical outcome measure in order to accurately assess the impact of treatment and the multidimensional factors affecting a patient's HR-QOL.

A systematic literature review of published studies of HR-QOL in GTS was conducted on the databases PubMed and PsycInfo, using 'Tourette', 'tics', and 'quality of life' as search terms. Full articles, including electronic early release publications, were obtained and references were checked for additional materials when appropriate. This literature review generated 13 studies which specifically addressed HR-QOL in individuals with GTS: 9 studies assessed HR-QOL in young patients and 4 studies focused on adults. These studies are summarized in Table 1 and discussed in the following paragraphs, in chronological order. Two further studies, dealing with the development and validation of the first disease-specific HR-QOL scale for GTS, will be presented in a separate section.

Elstner et al. interviewed 103 adults with a DSM-III-R-validated diagnosis of GTS from a specialist clinic (mean age 28.7 years; range 16–54 years), of whom 90 completed standardised measures of tic severity, OCD, depression and anxiety [31]. HR-QOL was assessed using a generic instrument, the Medical Outcomes Study 36-Item Short-Form Health Survey (SF-36) [32], comprising the following eight scales: physical functioning, role limitation due to physical functioning, bodily pain, social functioning, role limitation due to emotional problems, vitality, general health perception and mental health. The SF-36 is complemented with a self-evaluation of change in health over the past year. HR-QOL was also evaluated using the Quality of Life Assessment Schedule (QOLAS), a generic, patient-driven approach to HR-QOL assessment, based on the personal construct theory and repertory grid technique [33]. Based on these HR-QOL measures, patients with GTS showed significantly worse HR-QOL than a general population sample, but better HR-QOL than patients with intractable epilepsy. According to multiple comparison analysis, factors influencing HR-QOL domains in this GTS sample were employment status, tic severity, OCB, anxiety and depression. The authors concluded that HR-QOL in patients with GTS is impaired and therefore suggested that self-report HR-QOL measurements should be used alongside conventional outcome measurements in assessing treatment efficacy.

Storch et al. examined HR-QOL in 59 children and adolescents (mean age 11.4 years; range 8–17 years) diagnosed with GTS or chronic tic disorder and consecutively referred to a specialist clinic [28]. Of the 59 youths, 16 had one co-morbid disorder and 27 had multiple co-morbidities. Co-morbid diagnoses were ADHD ($n = 28$), OCD ($n = 25$), major depressive disorder ($n = 6$), generalized anxiety disorder ($n = 7$), oppositional defiant disorder ($n = 6$), Asperger syndrome ($n = 3$), social phobia ($n = 2$) and panic disorder ($n = 1$). For all subjects, the authors collected tic severity scores and parent-rated measures of the frequency and intensity of behavioural and emotional problems exhibited by their children (classified as 'internalizing' and 'externalizing' problems). Moreover, each patient completed the Pediatric Quality of Life inventory (PedsQL), a 23-item HR-QOL measure consisting of four independent domains (physical, emotional, social and school functioning) combined to yield physical (equivalent to the physical functioning domain only), psychosocial (sum of emotional, social, and school functioning domains) and total health scales (sum of all four domains) [34]. The patients' parents were asked to complete the PedsQL-Parent Proxy version. This study yielded four main findings. First, children with tics experienced lower HR-QOL than non-clinical samples in all areas except physical functioning, demonstrating that the presence of tics has a negative impact on children's lives. Specifically, children with tics rated their HR-QOL similarly to children with other psychiatric disorders. Second, overall tic severity was inversely associated with HR-QOL, possibly because tics have a direct impact on physical well-being and/or draw negative attention by others. Third, parent-child agreement in HR-QOL reports was stronger for the younger children (8–11 years) whereas these correlations were generally weaker for youths aged 12–17 years. This difference could be explained by the fact that parents of younger children may spend more time with their children and/or communicate more regarding their HR-QOL. Finally, internalizing and externalizing behavioural symptoms were significantly relat-

Table 1

Summary of published studies on HR-QOL in GTS populations

| Study | Country | N | Mean age (range); Sex | Diagnosis | Setting | HR-QOL measure (type) | Main findings | Main limitations |
|---|---|---|---|---|---|---|---|---|
| Elstner et al., 2001 [31] | UK | 103 | 29 (16–54) 70 M | GTS (21.0% GTS + OCD) | University-based clinic for GTS | QOLAS (interview-based) SF-36 (self-report) | 1.HR-QOL in GTS was poorer than in the general population but better than in the epilepsy population (normative data) 2.Depression, anxiety, OCB, tic severity and employment status were associated with poor HR-QOL | 1.Referral bias 2.Limitations of instruments (qualitative and quantitative measures) 3.Potentially different coping strategies by long-standing and newly diagnosed patients |
| Storch et al., 2007 [28] | USA | 59 | 11 (8–17) 41 M | GTS/chronic tic disorder (47.5% GTS + ADHD, 42.4% GTS + OCD) | University-based clinic for tic disorders | PedsQL (self-report) PedsQL-Parent Proxy (parent-report) | 1.Tic severity and 'internalizing' and 'externalizing' behavioural symptoms significantly correlated with poor HR-QOL 2.Parent-child agreement in HR-QOL rating was stronger in children (age 8–11) than adolescents | 1.Sample size 2.Referral bias 3.Limitations of instruments |
| Bernard et al., 2009 [35] | USA | 56 | 10 (5–17) NA | GTS (64.3% GTS + ADHD, 8.9% GTS + OCD) | University-based clinic for neurological disorders | TACQOL-PF (parent-report) | 1.OCD and ADHD (inattentive subtype) significantly correlated with poor HR-QOL 2. Tic severity did not correlate with HR-QOL | 1.Sample size 2.Referral bias 3.Limitations of instruments (no self-report measure) |
| Cutler et al., 2009 [37] | UK | 57 | 11 (8–17) 46 M | GTS (31.6% GTS + ADHD, 17.5% GTS + OCD) | University-based clinic for GTS | PedsQL (self-report) | 1.HR-QOL in GTS was poorer than in the general population (normative data) 2.Tic severity, OCB and ADHD correlated with poor HR-QOL | 1.Sample size 2.Referral bias 3.Limitations of instruments |
| Pringsheim et al., 2009 [38] | Canada and USA | 71 | 11 (7–17) 56 M | GTS (56.3% GTS + ADHD, 40.8% GTS + OCD) | University-based clinics for GTS | CHQ (self-report) | 1.HR-QOL in GTS was poorer than in the general population, esp. in the family activities domain (normative data) 2.Patients with co-morbid conditions reported poorer HR-QOL than patients with 'pure' GTS | 1.Sample size 2.Referral bias 3.Limitations of instruments |
| Hao et al., 2010 [39] | China | 424 (+618 MI +250 EP +42 LE +1583HC) | NA (8–12) 293 M | GTS (NA) | Two triple-A pediatric hospitals | PedsQL 4.0 (self-report) | 1.HR-QOL in GTS was poorer than in HC but better than in LE 2.GTS, MI, EP provided similar ratings for overall HR-QOL 3.GTS reported better social functioning, but worse emotional/school functioning than MI/EP | 1.Referral bias 2.Limitations of instruments 3.No control for co-morbidities |
| Müller-Vahl et al., 2010 [40] | Germany | 200 | 35 (18–75) 150 M | GTS (NA) | Three university-based clinics for GTS | EQ-5D (self-report) | 1.HR-QOL in GTS was poorer than in the general population (normative data) 2.Depression, tic severity and age correlated with poor HR-QOL | 1.Referral bias 2.Limitations of instruments 3.No control for co-morbidities |
| Eddy et al., 2011 [43] | Italy | 50 | 13 (10–17) 44 M | GTS (52.0% GTS + ADHD, 48.0% GTS + OCD) | University-based clinic for GTS | YQOL-R (self-report) | 1.Depression, OCD and ADHD correlated with poor HR-QOL 2.Tic severity did not correlate with HR-QOL | 1.Sample size 2.Referral bias 3.Limitations of instruments 4.Variables accounted for limited amount of variance in HR-QOL score |

Table 1, continued

| Study | Country | N | Mean age (range); Sex | Diagnosis | Setting | HR-QOL measure (type) | Main findings | Main limitations |
|---|---|---|---|---|---|---|---|---|
| Eddy et al., 2011 [44] | Italy | 50 (+ 50 EP +102 HC) | 13 (10–17) 44 M | GTS (52.0% GTS + ADHD, 48.0% GTS + OCD) | University-based clinic for GTS | YQOL-R (self-report) | 1.HR-QOL in GTS was poorer than in the general population in specific contextual items, but not in perceptual items<br>2.The type of co-morbidity determined which HR-QOL domains were most affected (patients with 'pure' GTS scored lower in the environment domain; patients with GTS+OCD scored lower in the self and relationships domain) | 1.Sample size<br>2.Referral bias<br>3.Limitations of instruments |
| Conelea et al., 2011 [45] | USA | 232 | 12 (10–17) 192 M | GTS/chronic tic disorder (37.1% GTS + ADHD, 39.2% GTS + OCD) | Internet-based survey | PedsQL-SF15 (self-report) | 1.Tic severity, OCB and ADHD correlated with poor HR-QOL | 1.Selection bias (Internet access required)<br>2.Limitations of instruments (diagnoses of tic disorders/co-morbidities could not be confirmed)<br>3.Parental involvement may have affected youth responses |
| Lewin et al., 2011 [46] | USA | 500 | > 18 | GTS/chronic tic disorder (NA) | Internet-based survey | PQOL (self-report) | 1.Tic severity correlated with poor HR-QOL<br>2.The correlation between tic severity and functional impairment was stronger in the presence of anxiety or depression | 1.Selection bias (Internet access required)<br>2.Limitations of instruments (YGTSS designed to be administered by a clinician rather than self)<br>3.No control for co-morbidities |
| Conelea et al., 2012 [47] | USA | 672 | 35,5 (18–77) 399 M | GTS/chronic tic disorder (34.5% +OCD, 22.8% +ADHD, 27,8% +Mood Disorder, 22,6% +Anxiety Disorder ) | Internet-based survey | PQOL (self-report) | 1.Tic severity correlated with poor HR-QOL, psychological difficulties and greater disability<br>2.Positive correlation between tic severity and functional impairment 3.Social/public avoidance and experiences of discrimination result from tics | 1.Selection bias (Internet access required)<br>2.Limitations of instruments (YGTSS designed to be administered by a clinician rather than self) |
| Cavanna et al., 2012 [48] | UK | 46 | 23,9 (16–41) 41 M | GTS (54.3% GTS + ADHD, 30.4% GTS + OCD | University-based clinic for GTS | GTS-QOL (self-report) | 1.Tic severity, premonitory urges and family history of GTS identified as predictors during childhood of a poorer HR-QOL in adults with GTS | 1.Sample size<br>2.Referral bias |

Abbreviations: CHQ, Child Health Questionnaire; EP, epilepsy; GTS, Gilles de la Tourette syndrome; HC, healthy controls; HR-QOL, Health related quality of life; LE, leukemia; MI, migraine; NA, not available; OCB, Obsessive-compulsive behaviors; PedsQL, Pediatric Quality of Life Inventory; PQOL, Perceived Quality of Life Scale; QOLAS, Quality of Life Assessment Schedule; SDS, Sheehan Disability Score; SF-36, Medical Outcomes Study 36-Item Short-Form Health Survey; TACQOL-PF, TNO-AZL Children's Quality of Life scale-Parent Form; TSA, Tourette Syndrome Association; YGTSS, Yale Global Tic Severity Scale; YQOL-R, The Youth Quality of Life Instrument-Research version; GTS-QOL, Gilles de la Tourette Syndrome-Quality of Life Scale.

ed to both child- and parent-rated HR-QOL. However, parent-rated HR-QOL scores were generally lower in children with high externalizing symptoms than in children with low externalizing symptoms, thus showing that parents are likely to view externalizing symptoms as more problematic and disruptive than tics.

Bernard et al. assessed the association between tic severity, OCD, ADHD subtypes and HR-QOL in 56 young outpatients with a diagnosis of GTS (mean age 10 years; range 5–17 years) [35]. In this sample, 36 patients had co-morbid ADHD and 5 patients had co-morbid OCD. For each patient, tic severity, OCD and ADHD ratings were collected. The patients' parents also completed the TNO-AZL Children's Quality of Life scale-Parent Form (TACQOL-PF) [36], a generic HR-QOL measure with 56 items, covering seven domains: Physical Complaints, Motor Functioning, Autonomy, Cognitive Functioning, Social Functioning, Positive Emotions, Negative Emotions. In the TACQOL-PF, parents are asked to answer questions about these domains from the perspective of their child. Multiple regression was used to assess the relationship between tic/OCD/ADHD ratings and HR-QOL. According to the results of univariate analysis, tic severity ratings were not associated with HR-QOL. On the other hand, both ADHD and OCD were significantly related to HR-QOL. Specifically, sub-analysis of ADHD subtypes showed that inattentiveness, but not hyperactivity, was associated with a lower HR-QOL. When tic severity, OCD and ADHD were considered simultaneously, tic severity remained non-significant, whereas both ADHD and OCD ratings were statistically significant, indicating that these co-morbid conditions were both independent contributors to poorer HR-QOL. The authors concluded that HR-QOL in children with GTS relates primarily to co-morbidities of OCD and ADHD (predominantly inattentive symptoms), which should be prioritised when planning treatment strategies.

Cutler et al. investigated the effect of GTS on the lives of young patients, using both quantitative and qualitative techniques [37]. Their sample of 57 children (mean age 11.4 years; age range 8–17) was recruited from a specialist clinic. Co-morbid ADHD was diagnosed in 18 patients, whilst 10 patients had co-morbid OCD. The PedsQL was used to assess HR-QOL. Data analysis revealed that symptom severity (defined as the number and frequency of tics and the presence of OCD and ADHD symptoms) was correlated with a poor HR-QOL. The qualitative analysis yielded four main themes: the need for managing tic symptoms has a negative impact on a child's HR-QOL; issues with being socially accepted and conforming to norms are seen as a challenge; the continuous struggle to control tics to avoid embarrassment causes subjective distress; finally, the acceptance of GTS as part of what makes the children who they are can pose significant problems. The clinical implications of this study include both targeting co-morbidities for intervention and recognising that encouraging children to accept their condition could minimize emotional distress.

Pringsheim et al. investigated how children with GTS, with or without co-morbidities (ADHD and/or OCD), experience disability [38]. Clinicians compiled baseline information and symptom severity rating scales, whilst parents completed a generic health status measure designed specifically for children to assess limitations, functional impairment and participation restrictions affecting both physical and psychosocial health. Seventy-one children (mean age 11 years; age range 7–17) recruited at two specialist clinics were assigned to four subgroups: GTS only ($n$ = 20), GTS + ADHD ($n$ = 22), GTS + ADHD + OCD ($n$ = 18), and GTS + OCD ($n$ = 11). Almost all psychosocial domain scores were significantly lower than national norms for the GTS + ADHD and GTS + ADHD + OCD subgroups, whilst for the GTS only subgroup, only the family activities domain was significantly affected. A multiple linear regression model including diagnosis, age, sex, and GTS/OCD/ADHD symptom severity found that the most significant predictor of the psychosocial summary score was ADHD symptom severity. The authors concluded that children with GTS + ADHD $+/-$ OCD experience impairment in all aspects of psychosocial health.

Hao et al. tested the psychometric properties of the Chinese version of the PedsQL 4.0 generic core scales on a relatively large sample of paediatric patients with acute and chronic conditions encompassing GTS, migraine, epilepsy, leukemia ($n$ = 1335) and healthy controls ($n$ = 1583) [39]. The results of this study showed that children with GTS, migraine and epilepsy rated their overall HR-QOL as better than patients with leukemia, but worse than healthy controls. Within the psychosocial domain, children with GTS reported better social functioning, but worse emotional or school functioning, than patients with migraine or epilepsy. According to the authors, their findings provided reasonable evidence to show that the Chinese PedsQL 4.0 has acceptable psychometric properties, with the exception of construct validity (tested by confirmatory factor analysis) and internal reliability for self-report in the paediatric samples with GTS and migraine.

Müller-Vahl et al. investigated the correlates of HR-QOL in an adult population of patients with GTS in Germany [40]. They assessed 200 patients (mean age 34.9 years; age range 18–75) using one of the most widely used generic measures of HR-QOL, the EQ-5D. The EQ-5D consists of five dimensions (Mobility, Self-Care, Usual Activities, Pain/Discomfort and Anxiety/Depression), which are rated on a three-point scale: no problems, some problems and extreme problems. A visual analogue score (EQ-VAS) is also incorporated into the final EQ-5D index score [41]. The EQ-5D has proven useful for the assessment of HR-QOL across movement disorders characterized by neuropsychiatric symptoms, such as Parkinson disease [42]. The authors of this study found that increased tic severity, patient's age and co-occurring depression were associated with a poorer HR-QOL. However, there were no data available on co-morbid ADHD or OCD.

Eddy et al. enrolled 50 young people (mean age 13 years; age range 10–17) with a DSM-IV-TR validated diagnosis of GTS to investigate the clinical correlates of HR-QOL [43]. These authors administered a battery of six standardized psychometric measures along with a generic HR-QOL questionnaire, the Youth Quality of Life Instrument-Research version (YQOL-R), consisting of 'perceptual' and 'contextual' items. The 41 perceptual items address the patients' perception of their HR-QOL across four specific domains: the self domain, the relationships domain, the environment domain and the general QOL domain. These items tackle issues ranging from how patients feel about themselves, to their relationships with peers and the world they live in. The 15 contextual items are of a more objective nature, relating to specific aspects of life that can affect HR-QOL. Multiple stepwise regression analysis revealed that the measures assessing the presence of OCD, ADHD and depression were the strongest predictors of HR-QOL. Interestingly, tic severity was not significantly associated with HR-QOL.

Eddy et al. also conducted the only controlled study specifically addressing HR-QOL in GTS to date, comparing the HR-QOL of young people with GTS with that of patients with epilepsy and healthy subjects [44]. In light of the controversy in previous research, these authors further examined whether tic severity or the presence of co-morbidities has a greater bearing on perceived HR-QOL. The YQOL-R was used to assess HR-QOL, as in their previous study. A total of 202 young people were recruited (mean age 12.9 years; age range 10–17) and divided into three groups: patients with GTS ($n = 50$), patients with benign idiopathic epilepsy ($n = 50$) and healthy controls ($n = 102$). Within the GTS group, patients were subdivided according to presence of co-morbid conditions: patients with 'pure' GTS (22%), patients with GTS and ADHD (30%), patients with GTS and OCD (26%) and patients with both co-morbidities (22%). The patients in the GTS group were also classified into two groups according to the severity of their tics, as measured by the Yale Global Tic Severity Scale (YGTSS). The subgroups were independently compared to the patients with epilepsy and the healthy controls for the HR-QOL scores. Increased tic severity and the presence of co-morbidities were associated with a poor HR-QOL. Moreover, the presence of different co-morbidities influenced which domains of HR-QOL were most affected. For example, patients with 'pure' GTS scored lower in the environment domain, whilst patients with co-occurring OCD scored lower in the self and relationships domain. These difficulties were more pronounced in patients with GTS compared to those with epilepsy or healthy controls. Patients with 'pure' GTS also exhibited more depressive symptoms compared to both patients with epilepsy and controls.

Conelea et al. conducted a large internet-based survey involving 740 parents with children with chronic tic disorders (CTD) and 232 children in the youth sample (mean age 12 years; age range 10–17) [45]. The Tourette Syndrome Impact Survey for Children (TSIS-C) was developed specifically for this study in order to gather data on the functional impact of living with CTD from both parent and child perspectives. The parent and youth sections of the survey-generated data could be classified into five domains: physical, social, familial, academic, and psychological. The authors used the PedsQL to evaluate HR-QOL, along with a standardized battery of questionnaires to assess tics, anxiety, depression and family impact. According to the presence of co-morbid conditions, the sample was categorized into CTD-Only patients (parent sample CTD-Only: $n = 361$, youth sample CTD-Only: $n = 107$) and CTD-Plus patients (parent sample CTD-Plus: $n = 375$, youth sample CTD-Plus: $n = 125$). Co-morbid behavioural problems included: ADHD, OCD, anxiety disorders, depression, disruptive behaviour disorders, bipolar disorder, somatoform disorder, learning disabilities, eating disorders, pervasive developmental disorders, trichotillomania and mental retardation. In accordance with previous studies, both increased tic severity and the presence of co-morbid psychiatric disorders were associated with a poorer HR-QOL and greater functional impairment. Moreover, the authors found that children with GTS experience marked discrimination due to their tics. Anxiety, depression and family functioning difficulties were also found to be more prevalent in this population than in the general population.

Lewin et al. expanded on the work by Conelea et al. in their internet-based survey of 500 adult patients with CTD to investigate whether the relationship between tic severity and both functional impairment and QOL was moderated by the presence of anxiety and depression [46]. The authors found a strong correlation between tic severity and functional impairment, especially in patients suffering from anxiety and depression. Conversely, where patients scored less on anxiety and depression scales, there was a weaker association between tic severity and functional impairment. It was suggested that a combination of therapies targeting both tics and anxiety/depression may improve outcomes.

Conelea et al. explored the functional impact of tics in adults using a sample of 672 participants with a self-reported CTD (mean age 35,5 years; range 18–77 years). They assessed the impact of tics on physical, social, occupational/academic, and psychological functioning using the Tourette Syndrome Impact Survey (TSIS Scale) and the Sheehan Disability Scale (SDS). Global functioning and quality of life was also evaluated using the PQOL. Results suggested mild to moderate functional impairment and positive correlations between tic severity and impairment. Notable portions of the sample reported also social or public avoidance and experiences of discrimination resulting from tics. Compared to previously reported population norms, participants had more psychological difficulties, greater disability, and poorer quality of life. The presence of motor tics interfering with voluntary behaviour was identified as the strongest predictor for disability, QOL and psychological difficulties [47].

Finally, Cavanna et al. investigated the childhood predictors of HR-QOL in a cohort of adult patients with GTS. In this study 46 patients with GTS aged 6 to 16 years underwent a baseline standardised clinical assessment of both tics and behavioural symptoms at a specialist GTS clinic. The same patients were reassessed aged 16 years and above, with a mean follow-up period of 13 years (range 3–25 years), using the Gilles de la Tourette Syndrome – Quality of Life Scale (GTS-QOL), a disease-specific measure of HR-QOL. The results of the multiple linear regression analysis showed that tic severity, premonitory urges and family history of GTS could be identified as predictors during childhood of a poorer HR-QOL in adults with GTS [48].

## 3. The GTS-QOL: A disease-specific HR-QOL measure

Albeit heterogeneous in methodology and sample sizes, the reviewed studies show that HR-QOL is emerging as a critical measure of clinical outcome as it takes into account the patient's own subjective view [49]. Disease-specific HR-QOL measures have been developed for patients suffering from neuropsychiatric conditions bearing similarities to GTS, including cervical dystonia [50] and hemifacial spasm [51]. However, until recently, there has been no tool to measure GTS-specific HR-QOL.

The GTS-QOL [52] was developed to fill this gap. The development and validation process, which included a large field-test on a sample of 136 patients recruited through the UK Tourettes Action, resulted in a clinically meaningful and psychometrically sound 27-item instrument for measuring HR-QOL in adults with GTS. Specifically, the scale satisfied standard criteria for acceptability, convergent and discriminant validity, internal consistency and test-retest reliability. The items of the GTS-QOL are grouped into four domains (psychological, physical, obsessive-compulsive and cognitive) and the instrument is complemented with a visual analogue scale (VAS) rating overall satisfaction with life. The domains and items of the GTS-QOL are shown in Table 2.

The GTS-QOL has the potential to complement both clinical rating scales and generic HR-QOL measures. These two types of traditional assessments have significant limitations as clinical tools and outcome measures. By definition, assessment of disease severity using clinical rating scales omits patient views about issues of importance to their health, particularly emotional and cognitive functioning and the subjective impact of dysfunction on daily life. Of note, it has been shown that perception of patients' HR-QOL by physicians and patients themselves can substantially diverge from each other [53]. Generic HR-QOL instruments do not incorporate, and are unlikely to be sensitive to specific features which are central to patients with GTS (motor and vocal tics, tic-related symptoms), and are likely to underestimate health problems in GTS. Therefore, the GTS-QOL may prove useful not only to evaluate HR-QOL in individual patients but also in epidemiological and observational trials and to relate neuroimaging or pathophysiological findings to subjective perception of HR-QOL. In clinical practice, this instrument holds promise as an outcome measure in longitudinal studies and clinical trials of treatment strategies, including

Table 2
Domains and items of the disease-specific Gilles de la Tourette Syndrome-Quality of Life scale (GTS-QOL)

| Domain | Items |
|---|---|
| Psychological | – Anxiety<br>– Depressed mood<br>– Difficulty seeing friends<br>– Frustration<br>– Lack of control over own life<br>– Lack of self-confidence<br>– Lack of social support<br>– Loneliness/isolation<br>– Mood switches<br>– Restlessness<br>– Temper discontrol |
| Physical/ADL | – Difficulty in activities of daily living<br>– Difficulty taking part in social activities<br>– Embarrassing gestures<br>– Involuntary swearing<br>– Movement discontrol<br>– Pain or injuries<br>– Phonic tics |
| Obsessive-compulsive | – Concerns about poor health<br>– Copying people<br>– Repeating actions<br>– Repeating words<br>– Unpleasant thoughts |
| Cognitive | – Difficulty concentrating<br>– Difficulty finishing tasks<br>– Losing important things<br>– Memory problems |

behavioural therapies, medications and neurosurgical procedures.

The GTS-QOL has recently been adapted to the child and adolescent population with GTS [54]. The process of back translation, language adaptation and scale validation has led to the development of two instruments, which are suitable for use in children and adolescents with GTS, respectively (GTS-QOL-C&A). The psychometric properties of these instruments have proven satisfactory in a field test on children and adolescents recruited at three special GTS centres in Italy. The validation of the GTS-QOL-C&A in English language is currently ongoing.

## 4. Preliminary conclusions and suggestions for future research

GTS is a lifelong disorder which causes functional impairment, disability, and psychological distress. The results of the reviewed studies suggest that the broad clinical picture of GTS should be addressed when assessing the patient's and family's perception of its impact on HR-QOL. Specifically, the severity of tics and presence of co-morbidities, particularly OCD and ADHD, are associated with a poorer HR-QOL in children. It is therefore important that clinicians routinely assess patients to determine which aspects of HR-QOL are most affected are therefore require attention regarding planning treatment. A similar picture exists for adults, where depression and anxiety also contribute to overall HR-QOL. Disease-specific instruments, such as the GTS-QOL, capture all relevant aspects of HR-QOL and thus should be regarded as important outcome measures for therapeutic interventions and useful tools in developing appropriate care plans for patients with GTS. Importantly, the GTS-QOL includes a subjective patient perception of HR-QOL which can be interpreted in light of the objective disability scores in order to help plan patient-centered care.

This paper reviewed the recent literature on HR-QOL in GTS, spanning one decade. The reviewed evidence has significant limitations as the studies differ in research methodology. For example, different instruments were used to assess HR-QOL, none of which were disease-specific; therefore, the results of each study may be skewed to the focus of a given instrument. The means of data collection differs vastly from clinical series to internet surveys and therefore this review is limited in the cohesiveness with which the results can be collated. It is also unclear how the studies accurately diagnose co-morbidity and whether the criteria for such a diagnosis are comparable across different studies. Furthermore, participants involved in the studies were mostly recruited from tertiary referral centres, which attract more complex cases, often with multiple co-morbidities. As a consequence of referral bias, the study samples may not be a generalizable representation of the community population of patients with GTS and thus may not reflect the influences on HR-QOL in the average patient. Some of the studies used proxy HR-QOL measures whereby parents rated their child's HR-QOL, thus introducing a further accuracy bias [15]. Storch et al., for example, showed that the parent-child agreement in the adolescent group was poorly correlated [28]. The paucity of studies in children and adolescents presents a problem since this disorder has its onset in childhood. The issue of both lack of childhood studies and small sample sizes could be addressed in future research by studying a community sample and reporting on the HR-QOL from a truly large sample across all ages. Furthermore, biases within the sample exist due to limitations in access to care and the relative paucity of practitioners who are skilled in diagnosing and treating systems of the disorder in ways that are supported by evidence.

Analysis of the first decade of studies specifically addressing HR-QOL in GTS suggests some general directions for future research. An interesting preliminary observation is that whilst co-morbid conditions appear to play a central role in determining HR-QOL in younger patients, the picture is more complex in adults with GTS. Possible reasons for this trend could be the natural course of common neurodevelopmental comorbidities such as ADHD, which spontaneously improves with age, and the development of copying strategies for behavioural problems through adolescence and early adulthood. Ascertainment bias could also be partly responsible for these observations, as adult patients attending specialist clinics for GTS, rather than general psychiatry clinics, are more likely to be seeking help for the management of severe tics. These findings offer some general directions for both current clinical practice and future research. Cross-sectional studies are now abundant and therefore future research needs to focus on longitudinal studies to determine how the natural history of GTS and patient adaptation and adjustment may influence changes in HR-QOL from childhood to adulthood. Future studies might build on what has been learned from the limitations of each of the studies described here and how these papers have moved the field forward. Future research should also explore the use of the newly developed disease-specific measures of HR-QOL (GTS-QOL and GTS-QOL-C&A) for the assessment of both clinic and community patients with GTS.

## Acknowledgments

The authors are grateful to Tourette Syndrome Association-USA and Tourettes Action-UK for continuing support. VB was funded by COST-Action BM0905.

## Conflicts of interest

The authors have no conflicts of interest to declare.
